# Correction: Thenuwara et al. Recent Advancements in Lateral Flow Assays for Food Mycotoxin Detection: A Review of Nanoparticle-Based Methods and Innovations. *Toxins* 2025, *17*, 348

**DOI:** 10.3390/toxins17100505

**Published:** 2025-10-14

**Authors:** Gayathree Thenuwara, Perveen Akhtar, Bilal Javed, Baljit Singh, Hugh J. Byrne, Furong Tian

**Affiliations:** 1 School of Food Science and Environmental Health, Technological University Dublin, Grangegorman, D07 ADY7 Dublin, Ireland; d24127925@mytudublin.ie (G.T.); d24127942@mytudublin.ie (P.A.); baljit.singh@tudublin.ie (B.S.); 2 Nanolab Research Centre, Physical to Life Sciences Research Hub, Technological University Dublin, Camden Row, D08 CKP1 Dublin, Ireland; 3 MiCRA Biodiagnostics Technology Gateway and Health, Engineering & Materials Science (HEMS) Research Hub, Technological University Dublin, D24 FKT9 Dublin, Ireland

Figure/Legend

In the original publication [1], there was a mistake in the figure and legend of “Figure 1. Schematic of a competitive lateral flow assay (LFA) for mycotoxin detection”. “A sandwich immunoassay design was included for the schematic of a LFA”. The correct figure and legend appear below. The authors state that the scientific conclusions are unaffected. This correction was approved by the Academic Editor. The original publication has also been updated.

**Figure 1:**
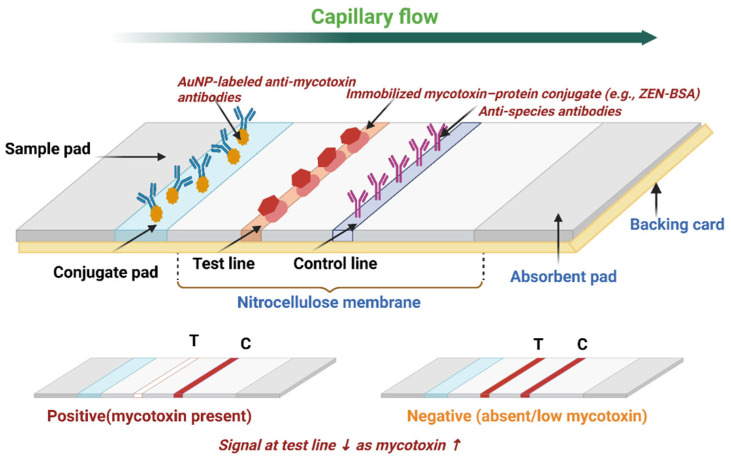
Schematic of a competitive lateral flow assay (LFA) for mycotoxin detection. The assay consists of a sample pad, conjugate pad, nitrocellulose membrane with test (T) and control lines (C), absorbent pad, and backing card. AuNP-labeled anti-mycotoxin antibodies migrate with the sample by capillary flow and interact either with free mycotoxin analytes (hapten) in a solution or with the immobilized mycotoxin–protein conjugate (e.g., ZEN–BSA) at the test line. In the presence of mycotoxin, free analytes compete for antibody binding, resulting in a reduced or absent signal at the test line, while the control line remains visible, confirming assay validity and a positive result. The signal intensity at the test line is inversely proportional to the mycotoxin concentration in the sample (bottom left). In contrast, when the mycotoxin is absent, antibody–nanoparticle conjugates bind to the immobilized conjugate, generating a visible signal at the test line alongside the control line, indicating a negative result (bottom right). Red arrows indicate that signal intensity at the test line decreases as mycotoxin concentration increases (created with Biorender).

In the original publication, there was a mistake in the figure and legend of “Figure 2: Schematic representation of label designs”. “A sandwich format was included for the signal amplification of LFA”. The correct figure and legend appear below.

**Figure 2:**
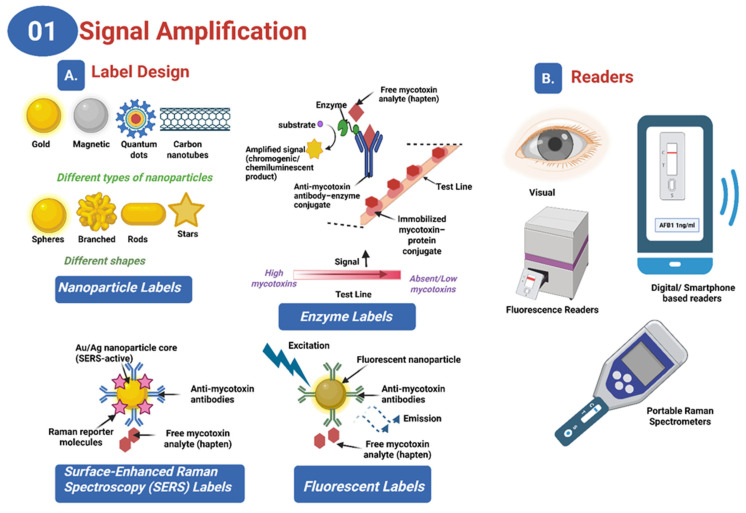
Schematic representation of label designs (**A**) and detection readers (**B**) used for signal amplification in lateral flow assays for mycotoxin detection. Labeling strategies include nanoparticles, enzymes, surface-enhanced Raman spectroscopy (SERS), and fluorescent labels. Reader technologies range from visual inspection to portable optical, and Raman devices, with smartphone-based digital readers offering field-deployable quantification (created with Biorender).
